# Novel Use of Hydroxyurea in an African Region With Malaria: Protocol for a Randomized Controlled Clinical Trial

**DOI:** 10.2196/resprot.5599

**Published:** 2016-06-23

**Authors:** Juliana N Anyanwu, Olatundun Williams, Casey L Sautter, Phillip Kasirye, Heather Hume, Robert O Opoka, Teresa Latham, Christopher Ndugwa, Russell E Ware, Chandy C John

**Affiliations:** ^1^ University of Minnesota Minneapolis, MN United States; ^2^ Northwestern University Chicago, IL United States; ^3^ Makerere University Kampala Uganda; ^4^ University of Montreal Montreal, QC Canada; ^5^ Cincinnati Children's Hospital Medical Center Cincinnati, OH United States; ^6^ Indiana University School of Medicine Indianapolis, IN United States

**Keywords:** sickle cell anemia, hydroxyurea, malaria

## Abstract

**Background:**

Sickle cell anemia (SCA), one of most prevalent monogenic diseases worldwide, is caused by a glutamic acid to valine substitution on the beta globin protein of hemoglobin, which leads to hemolytic anemia. Hydroxyurea, the only disease-modifying therapy approved by the Food and Drug Administration for SCA, has proven to be a viable therapeutic option for SCA patients in resource-rich settings, given clinical improvements experienced while taking the medication and its once-daily oral dosing. Significant studies have demonstrated its safety and clinical efficacy among children and adults in developed countries. In Sub-Saharan Africa, however, the risk of malaria, hematologic toxicities, and safety of hydroxyurea in children with SCA living in malaria-endemic areas are unknown.

**Objectives:**

Study objectives include determining the incidence of malaria in SCA patients taking hydroxyurea versus placebo; establishing the frequency of hematologic toxicities and adverse events (AEs) in children with SCA treated with hydroxyurea versus placebo; and defining the relationships between hydroxyurea treatment and fetal hemoglobin, soluble intracellular adhesion molecule-1, and nitric oxide levels, and between levels of these factors and risk of subsequent malaria.

**Methods:**

Novel use Of Hydroxyurea in an African Region with Malaria (NOHARM, NCT01976416) is a prospective, randomized, placebo-controlled, double-blinded phase III trial to compare risk of malaria with oral hydroxyurea versus placebo. Children will be recruited from the Mulago Hospital Sickle Cell Clinic in Kampala, Uganda.

**Results:**

Two hundred Ugandan children aged between 1.00 and 3.99 years with confirmed SCA will be randomized into treatment groups by order of entry in the study, based on a predetermined blinded randomization list. The primary outcome of the trial is malaria incidence in the 2 study groups, defined as episodes of clinical malaria occurring over the 1-year randomized study treatment period.

**Conclusion:**

NOHARM will be the first prospective randomized, placebo-controlled clinical trial investigating the use of hydroxyurea for children with SCA in a malaria-endemic region within Africa. The results of this trial have the potential to significantly advance understanding of how to safely and effectively use hydroxyurea in children with SCA in malaria-endemic areas.

**Trial Registration:**

Clinicaltrials.gov NCT01976416; https://clinicaltrials.gov/ct2/show/NCT01976416 (Archived by WebCite at http://www.webcitation.org/6hmoilZnp)

## Introduction

### Background and Rationale

Sickle cell anemia (SCA) is a genetic blood disorder where sickling of red blood cells, due to a genetic alteration in hemoglobin, leads to chronic hemolysis, organ damage, vaso-occlusive events, and other potentially life-threatening complications [1]. It is acquired in an autosomal recessive fashion and is most prevalent in Africa, where over 230,000 babies with SCA are born annually [2]. Without any identification by newborn screening or early interventions, many of these children will die [3,4] from acute anemia or infection, especially bacterial sepsis or malaria, and many before the age of 5 years. Because SCA is an important cause of under-5 mortality, the World Health Organization (WHO) has urged African nations to make its recognition and management a priority [5,6].

Despite being the most prevalent genetic disease in the African region, disease prevention and management for SCA are inadequate [5]. With the high prevalence and morbidity of SCA in Sub-Saharan Africa, there is need for improved access to diagnostic and therapeutic options. Stem cell transplant and blood transfusions are effective therapeutic considerations in high-income countries but often not feasible in low-income countries owing to limited availability and greater risks within these settings [7]. In many developed countries, interventions such as prophylactic penicillin, pneumococcal immunizations, newborn screening programs, and the availability of hydroxyurea have significantly decreased SCA-associated morbidity and mortality, thereby leading to improved survival into adulthood.

Hydroxyurea, an inducer of fetal hemoglobin (HbF), saw its first clinical application for SCA in 1984. The drug has been shown to dramatically reduce vascular crises, hospitalizations, and the need for transfusions among SCA patients. It is inexpensive, can be given with once-daily dosing, and has been shown to be safe and effective from the age of 9 months to adulthood [8,9]. Thus, it has been proposed as the standard of care for children in the United States with SCA [9].

At the time of this paper’s publication, however, hydroxyurea is not the standard of care in Sub-Saharan Africa for SCA, as the safety of the drug in a malaria-endemic region is not known. Studies have shown that children with SCA have higher mortality rates from malaria than children with either sickle cell trait (hemoglobin AS, HbAS) or children with normal hemoglobin. Children with HbAS are strongly protected from severe malaria [10], and this is thought to be the main reason why the hemoglobin S gene has been retained through selection in African populations living in malaria-endemic areas. Children with SCA appear to have a similar or lower risk of uncomplicated and severe malaria as children without SCA but have a much higher rate of death when hospitalized with malaria [11]. The reasons for the increased risk are not fully elucidated but likely relate to worsened anemia and/or sepsis from the gram-negative bacteremia that often occurs with severe malaria [12]. Thus, prevention of malaria is critical in children with SCA because severe malaria disproportionately leads to death in these children.

Many of the factors relating to vaso-occlusive crises and other complications in SCA are important in the pathogenesis of severe malaria. Factors associated with severe malaria that may be affected by hydroxyurea in children with SCA include intracellular adhesion molecule-1 (ICAM-1) [13,14], vascular cellular adhesion molecule-1 (VCAM-1) [15,16], von Willebrand factor (VWF) [17], tumor necrosis factor-alpha (TNF-α) [18], and nitric oxide (NO) [19]. All these factors, except NO, are elevated in severe malaria, and all are potentially involved in the pathogenesis of severe malaria. For example, elevated ICAM-1 expression in animal models of severe malaria leads to increased parasite adhesion and worsened clinical malaria [20], and our group and others have shown that children with severe malaria have elevated VWF levels, suggesting that endothelial activation in severe malaria may be associated with more severe disease [21]. Studies have found that TNF-α is elevated in children with severe malaria [22] and increased in children who die of malaria [22]. Animal studies suggest that TNF-α is directly involved in pathogenesis of severe malaria and is not simply a marker of disease severity [23]. In contrast, NO appears to be an important factor in protection against severe malaria in human [24] and animal [25,26] studies, through mechanisms including vasodilation and direct toxicity against the parasite.

Hydroxyurea is thought to prevent vaso-occlusive crises and pain episodes in children with SCA in part through generation of NO [19,27,28]. Through this mechanism, hydroxyurea could actually lead to protection from malaria. Similarly, HbF has been shown to inhibit *Plasmodium falciparum* growth *in vitro* [29] and is thought to be responsible for much of the protection from clinical malaria seen in children aged <3 months in malaria-endemic areas [30], and hydroxyurea typically increases (in older children) or slows the decrease (in children aged <18 months) of HbF levels. Both these changes should lead to *decreased* risk of malaria in children taking hydroxyurea. However, the effect of hydroxyurea on markers of endothelial activation is less clear. Early studies found that hydroxyurea raised concentrations of ICAM-1 in an *in vitro* model of SCA [13], raising concern that this could lead to an increased risk of parasite binding in children with SCA and therefore to increased sequestration and complications (including death) from malaria. However, one study in humans showed that hydroxyurea decreased levels of soluble ICAM-1 (sICAM-1) [15], and another study showed no change in sICAM-1[16]. A subsequent animal study did not show increased ICAM-1 expression or worse outcome with hydroxyurea treatment of animals with animal model SCA [14], but the animal models used a *Plasmodium* species other than *P. falciparum,* which may interact differently with ICAM-1 than *P. falciparum*. *In vitro* studies suggest that hydroxyurea decreases VWF incorporation into the endothelial cell extracellular matrix [17], but human studies show no change in VWF levels with hydroxyurea treatment [16]. Early studies of hydroxyurea in rats demonstrated that it increased TNF-α levels [31], but one human study of individuals with SCA receiving hydroxyurea treatment showed a decrease in TNF-α levels [18], and another showed no difference in TNF-α levels [32]. A recent review of malaria in children with sickle cell disease nicely outlined other ways in which hydroxyurea might combat malaria, including direct antiparasitic activity at high concentrations, and improvement in splenic function [33], but it is unclear whether these effects or an increase in ICAM-1 or TNF-α, which may increase risk of malaria severity, will be seen in children with SCA in Sub-Saharan Africa.

The hematologic toxicities of hydroxyurea, particularly neutropenia, are additional important safety concerns for children with SCA in malaria-endemic areas of Sub-Saharan Africa. The recently completed BABY-HUG study reported hematologic toxicities with hydroxyurea treatment that included severe neutropenia (5%), severe anemia (1%), and thrombocytopenia (11%), although similar toxicities were also noted in the placebo-treated arm [8]. Malaria can decrease bone marrow erythropoietic response and lower platelet count, so, the side effects of anemia and thrombocytopenia from hydroxyurea treatment could be greater in children repeatedly exposed to malaria than in children in North America or Europe who lack malaria exposure. Neutropenia in children in Sub-Saharan Africa may also place them at higher risk of severe bacterial infection than neutropenia in children in North America or Europe because of the much higher baseline rates of bacterial infection in African children, which could then lead to invasive and severe bacterial infection.

In summary, some changes associated with hydroxyurea treatment (increased NO and HbF and improved splenic function) would be expected to protect against malaria, but the data on hydroxyurea-related endothelial changes thought to be important in severe malaria pathogenesis (eg, ICAM-1, VWF, TNF-α) are less clear, with some studies suggesting that these factors might be increased with hydroxyurea and others suggesting no difference or even a decrease. The frequency, severity, and consequences of the hematologic toxicities of hydroxyurea in children with SCA in malaria-endemic areas are also unknown. Thus, a prospective clinical trial to determine definitively whether hydroxyurea poses special risks to children with SCA in malarial regions is warranted.

### Study Objectives and Hypotheses

The objectives of the study are:

1. To determine the incidence of malaria in children with SCA treated with hydroxyurea versus placebo: The working hypothesis of this aim is that incidence of malaria is not greater in children with SCA treated with hydroxyurea in comparison to those treated with placebo. We will test this hypothesis by comparing malaria incidence over a 1-year period in children with SCA in the hydroxyurea versus placebo treatment groups.

2. To establish the frequency of hematologic toxicities and AEs in children with SCA treated with hydroxyurea versus placebo: The working hypothesis of this aim is that children with SCA treated with hydroxyurea will have more medication-related hematologic toxicities, such as neutropenia, but no increase in SCA-related AEs (eg, pain crises, hospitalizations, requirement of blood transfusion) compared with children treated with placebo. We will test this hypothesis by comparing hematologic toxicities and AEs in children with SCA in the hydroxyurea versus placebo treatment groups.

3. To define the relationship between hydroxyurea treatment and HbF, sICAM-1, and NO levels and between levels of these factors and risk of subsequent malaria. The working hypotheses of this aim are that: (1) hydroxyurea will increase HbF and plasma NO levels and decrease plasma sICAM-1 levels and (2) HbF and plasma NO levels will inversely correlate, and plasma sICAM-1 levels will positively correlate, with subsequent malaria incidence. We will test this hypothesis by testing the associations between (1) hydroxyurea and change in levels of HbF, sICAM-1, and NO at 2-, 4-, and 12-month follow-up and (2) change in levels of HbF, sICAM-1, and NO at 2 and 4 months and risk of subsequent malaria.

### Trial Design

We are conducting a prospective, randomized, placebo-controlled, double-blinded phase III trial to compare oral hydroxyurea with placebo among Ugandan children with SCA. The trial opened to accrual in September 2014, and we estimate that the 2-year follow-up will be completed in December 2017.

## Methods

### Study Setting

Study participants will be recruited from the Mulago Hospital Sickle Cell Clinic (MHSCC) in Kampala, Uganda. The MHSCC is the first and largest specialized clinic for the treatment of SCA in Uganda. The clinic was established by Professor Christopher Ndugwa in 1968, and since its inception, over 11,000 SCA patients have been registered at the MHSCC. Currently, approximately 3,500 patients are actively treated at this clinic, about 20% of whom are aged younger than 4 years. Kampala is an area of mesoendemic malaria transmission, but the outpatient and inpatient malaria burden is still high. Malaria is the primary diagnosis in more than 2,500 children admitted annually to Mulago Hospital and more than 20,000 children seen as outpatients at Mulago Hospital.

### Eligibility Criteria

Children were recruited for the Novel use Of Hydroxyurea in an African Region with Malaria (NOHARM) study through the MHSCC Database of active patients, using the following criteria:

#### Inclusion Criteria

1. Pediatric patients with documented SCA (hemoglobin SS supported by hemoglobin electrophoresis or by peripheral blood smear showing sickled red blood cells).

2. Age range of 1.00-3.99 years, inclusive, at the time of enrollment.

3. Weight at least 5.0 kg at the time of enrollment.

4. Willingness to comply with all study-related treatments, evaluations, and follow-up.

Children who meet the aforementioned criteria, but who are acutely ill at the time of recruitment will be asked to return to clinic at a later date to establish baseline laboratory values and ensure that they do not meet any of the following exclusion criteria:

#### Exclusion Criteria

1. Active use of hydroxyurea on a regular basis.

2. Known chronic medical condition (eg, HIV, malignancy, active clinical tuberculosis).

3. Severe malnutrition determined by impaired growth parameters as defined by WHO (weight for length and height < −3 z-scores below the median WHO growth standards).

4. Preexisting severe hematologic toxicity: Hb <4.0 gm/dL, Hb <6.0 gm/dL with absolute reticulocyte count (ARC) <100 × 10^9^/L, ARC <80 × 10^9^/L with Hb <7.0 gm/dL, platelets <80 × 10^9^/L, and absolute neutrophil count (ANC) <1.0 × 10^9^/L.

5. Alanine aminotransferase or creatinine >2× the upper limit of normal for age.

6. Blood transfusion within 30 days before enrollment.

### Interventions

Medical evaluation and treatment for standard clinical issues will be performed as per the MHSCC guidelines. Control and treatment groups will receive the same standard care and will complete evaluations in the same time frame. Standard preventive measures provided in the MHSCC for children with SCA include the following: (1) folic acid (1 mg of PO) daily; (2) penicillin prophylaxis (250 mg PO) daily for all children aged younger than 5 years; (3) sulfadoxine-pyrimethamine prophylaxis, once monthly; and (4) anthelmintic treatment every 6 months. All children in the study will also receive an insecticide-treated bednet (ITN) because of the documented benefits of ITNs in population-based studies of malaria [34].

Eligibility screening and Baseline Evaluations will occur together at month 0. Study treatment initiation will also occur at month 0 or within 1 to 3 days of being found eligible for randomization. Study treatment will commence at 20 ± 2.5 mg/kg of PO daily (for all participants except those weighing 6.0-8.0 kg, in whom the dose will be closer to 20 ± 5mg/kg), with appropriate adjustments as needed for hematologic toxicities as outlined in the following section. The appropriate dose will be achieved using a combination of 100 mg and 1000 mg (4 × 250 mg scored) tablets.

#### Dose Initiation Plan for Study Months 0 to 12

Using the participant’s weight at month 0, the daily dose will be calculated using available tablet sizes and a goal of 20 ± 2.5 mg/kg/day using dosing tables based on the participant’s weight. At each interval visit, laboratory studies will be used to assess treatment toxicity, typically anemia or neutropenia, but possibly also reticulocytopenia or thrombocytopenia. The daily dose will be held or lowered based on protocol treatment toxicity guidelines. Medication adherence will be assessed at each visit, and attempts will be made to collect treatment compliance data.

#### Rationale for Placebo-Controlled Treatment

Due to the concerns about potentially increasing the risk of severe malaria with the use of hydroxyurea, a placebo-controlled randomized clinical trial is the only way the issue of hydroxyurea safety in this patient population can be resolved. The risk of death from malaria makes it imperative that the effects of hydroxyurea on malaria be studied in Ugandan children with SCA who are at risk of malaria. The study design was discussed and refined with local academic leaders before submission and approval by the Makerere School of Medicine Research Ethics Committee and the other institutional review boards (IRBs) listed in the following *Ethical Considerations* section.

### Outcomes

#### Specific Aim 1

##### Primary Outcome

The primary outcome of the NOHARM trial is malaria incidence, defined as episodes of clinical malaria occurring over the 1-year randomized study treatment period. Clinical malaria is defined as a history of fever or presence of measured fever (axillary temperature ≥37.5°C) in a child with a blood smear positive for *Plasmodium* species on microscopy. All parents or guardians are asked to bring their children to the MHSCC for any illness, and we anticipate compliance to be high because free evaluation and care by study personnel are available at the clinic. Children will be evaluated for malaria by a study clinician, and microscopy will be done by study personnel trained in malaria microscopy. All blood smears will be read independently by 2 readers, and any reading with a discrepancy between the 2 readers will have a third reading done to establish final diagnosis.

##### Secondary Outcomes

To assess incidence of malaria by additional criteria that may add specificity (at the cost of some sensitivity), we will add the following definitions as secondary outcomes. These definitions will include all children with the primary definition but with the added criteria of the following: (1) *P. falciparum* parasitemia >1000 parasites/µL; (2) measured axillary temperature ≥37.5°C; (3) *P. falciparum* parasitemia >1000 parasites/µL and measured axillary temperature ≥37.5°C; and (4) malaria requiring hospital admission. The following covariates will be assessed: (1) local council area or village (location of household where child lives), as a surrogate for malaria exposure. Malaria exposure in Kampala is heterogeneous. Randomization should effectively place children from different areas of exposure risk evenly in the 2 treatment groups, but we will assess this; (2) socioeconomic status (SES). We have developed a simple tool for evaluation of SES of the families of children in our malaria studies. SES is often related to malaria exposure; (3) age and gender.

#### Specific Aim 2

##### Primary Outcomes

The primary outcomes include: (1) composite outcome: one or more of the SCA-related AEs summarized in Table 1 (modified from the BABY-HUG trial) and (2) any hematologic toxicity noted in the *Dose Toxicity* section.

**Table 1:** Definitions of sickle cell anemia-related adverse events.

Adverse event	Definition
Pain event	Pain in the arms or legs, back, abdomen, chest, or head with no other explanation, lasting at least 2 hours, that brings child to clinic for evaluation and requires nonsteroidal anti-inflammatory or narcotic analgesics
Dactylitis	Pain and tenderness, with or without swelling, limited to the hands and feet
Acute chest syndrome	Clinical syndrome characterized by new pulmonary infiltrate and at least 3 of: chest pain, axillary temperature greater than 37.5°C, tachypnea, wheezing, or cough
Splenic sequestration	Increase in palpable spleen size by 2 cm or more below the costal margin from the last examination, accompanied by a decrease in hemoglobin of 2 g/dL or more or 20% or more from steady state values
Requirement of blood transfusion	Reason for transfusion will be recorded

##### Secondary Outcomes

The secondary outcomes include: (1) individual AEs, including painful events and (2) toxicities for hemoglobin, reticulocytes, neutrophils, or platelets. The following covariates will be assessed: age; gender; and weight and height for age, weight for height (nutrition).

#### Specific Aim 3

##### Primary Outcomes

The primary outcome will be change in percent HbF and plasma concentration of sICAM-1 and NO.

##### Secondary Outcomes

The secondary outcome will be change in concentration of plasma VCAM, VWF, and TNF. HbF levels and initial hemoglobin S testing will be performed by capillary electrophoresis (Sebia MINICAP) in the Uganda Cancer Institute laboratory. sICAM-1 and soluble VCAM-1 will be assessed by ELISA (R&D Systems), VWF activity by ELISA (Corgenix), and TNF-α by cytometric bead assay (EMD-Millipore). All these tests will be done in the University of Minnesota or University of California—San Francisco or Makerere University laboratory in Kampala, Uganda. NO levels will be measured by fluorometric assay of total plasma nitrate and nitrite (EMD-Millipore). NO levels will be tested at the Indiana University because a fluorometric assay reader is not available on the Makerere campus.

#### Participant Timeline

Subjects will have clinic visits and laboratory assessments at specific time points as detailed in Multimedia Appendix 1).

### Sample Size Calculation

We aimed to randomize 200 children with SCA into study arms (100 in each treatment arm). Twelve months of age was chosen as the minimum age based on published experience from the phase III infant hydroxyurea (BABY HUG) clinical trial [8]. An upper age limit of 4 years was chosen because older children have less common and less severe malarial events. Sample size calculations were completed for each specific aim as follows:

For Specific Aim 1, the detectable difference in malaria incidence depends on incidence in the control group. Few relevant data are available for children with SCA in Uganda, so, we present a range of detectable differences corresponding to a range of 0.3 to 1.3 malaria incidents per child in a year in the control group (based on data from an ongoing study of malaria in Kampala that involves assessment of malaria incidence in community children—lowest estimated incidence—and children with severe malaria—highest estimated incidence). With 100 children per group, these control-group rates imply 90% power to detect between 59% and 34% difference between groups, respectively in malaria incidence (This assumes a 2-sided test, alpha=0.05, and variance or mean=1.7 as in the ongoing study mentioned previously.)

For Specific Aim 2, the outcome of greatest interest for this aim is the occurrence of 1 or more AEs. We estimate that approximately 50% of control-group children will have 1 or more of the AEs summarized in Table 1, most frequently a pain event. The decision to treat 100 children per group provides 90% power to detect a difference from a rate of 73% in the hydroxyurea group (80% power for a rate of 70%). For the hematologic measures, we do not have estimates of the fraction of children who will reach the dose-limiting toxicities or the incidence of such events. Comparing the hydroxyurea and placebo groups according to average values of ANC or Hb, we have 90% power to detect a difference between groups of 0.46 standard deviations (ie, describing variation between children in the same group, hydroxyurea or placebo).

For Specific Aim 3, to test the association of study treatment with each marker, we have 80% power to detect a difference between groups of 0.40 standard deviations in the marker (this is conservative and is based on having a single follow-up marker measurement per child instead of the 3 we will have). To analyze the association between markers and malaria episodes, the detectable difference in rate of malaria episodes depends on the rate of episodes per child. For a low rate of 0.3 episodes per child in 10 months, we have 80% power to detect a rate ratio of 2.1 for a 1-standard-deviation change in the marker; for a high rate of 1.2 episodes per child, the detectable rate ratio is 1.5.

### Recruitment and Enrollment

Families of recruited subjects will be informed of study procedures and the expected follow-up schedule. Subjects will be enrolled by the study nurse or medical officer based on meeting inclusion criteria, not meeting exclusion criteria, and being able to maintain the follow-up schedule. Subjects will be enrolled after written informed consent has been obtained from the parent or guardian, according to the guidelines of the main IRB and the local ethics committees (Multimedia Appendix 2, 3 and 4). Given the burden of traveling to the clinic each month, remuneration per visit will be provided to families to help defray the costs of travel and lost wages.

### Randomization and Blinding

Block randomization will be used and will occur via the OnCore Electronic Data Capture system. OnCore is a software system widely used in many clinical research centers across the United States and internationally. The data coordinating center (DCC) will have uploaded randomization “block files” into the system so that participants can be randomized into the study.

Study participants will be randomized by the DCC staff into treatment groups by order of entry in the study, based on the predetermined blinded randomization list. The child’s study identification number will be recorded, and treatment group may only be determined by comparing the child’s study identification number to the blinded list, which only the DCC staff will have access to until the study is completed or stopping rules are reached and unblinding is required. The study pharmacist will have identical appearing hydroxyurea or placebo tablets and will provide the appropriate medication to the child. Dosage adjustment will be done according to the aforementioned guidelines so that if lower counts occur on placebo, placebo dose will also be lowered. Neither study participants nor their care providers, investigators, study personnel treating the participants, study coordinators, and outcomes assessors will know which arm a child is in. Laboratory values for scheduled visits are entered in the database and provided to the clinicians taking care of the study participant only if the laboratory value is a critical value.

### Data Management

Before site activation, the study investigators will ensure that adequate clinical, laboratory, pharmacy, data management facilities, and operations are in place. In addition, adequate training in all aspects of the trial, including electronic data entry, will be conducted. Research staff will undergo retraining as needed to ensure sound practices are maintained.

OnCore is Web based and password protected. The DCC has been using it for clinical trials management since 2009. Security privileges will be assigned in OnCore based on team roles and duties, within the context of the protocol. OnCore allows for validation of electronic case report forms, which allows data to be locked once they are verified.

Data from this study will be reviewed in real time by the study team members. Source documents will be reviewed, and data entries will be confirmed. Inconsistencies will be examined with the data manager, and corrective actions will be taken as needed. In regard to subject confidentiality, coded numbers will be used for identification of participant records and laboratory specimen. Clinical information will not be released by the NOHARM Medical Coordinating Center (MCC) or DCC without written permission from the subject, parent, or guardian, except as necessary for monitoring. Clinical information can be shared by the site investigators and staff for patient care reasons, for example, local consultations. The final trial dataset will be kept by the DCC, and no one outside the DCC, including the Principal Investigator, will have access to the randomization information or to the full trial dataset until the end of the randomized trial, unless findings in interim analysis lead the data safety and monitoring board (DSMB) to request study un-blinding. The DCC and MCC have a written agreement clarifying these roles.

Efforts for participant retention in the trial will be encouraged through the regular contact via visits for refill of hydroxyurea or placebo and provision of ongoing clinical care for the duration of the study. Outcome data will be recorded for those who withdraw from the study up to the time of withdrawal.

### Analysis

For *Specific Aim 1*, we will compare the hydroxyurea versus placebo groups according to total malaria incidence (inpatient and outpatient) over the 12-month treatment period, using an intention-to-treat analysis. The comparison will use negative binomial regression with adjustment for age (a known predictor of outcome), gender (if associated with outcome), local council area or village, and SES. This choice is based on analysis of preliminary data indicating significant overdispersion compared with Poisson-distributed counts. Secondary outcomes will be tested using similar methods. We will record whether a child was admitted for malaria, and the complication that led to admission, but the final number of episodes of malaria recorded as the primary outcome will be all episodes, whether treated as outpatient or requiring admission. Episodes requiring admission will be assessed in secondary analysis (see Secondary Outcomes, in the previous section).

*Specific Aim 2* is to establish the frequency of hematologic toxicities and AEs in children with SCA treated with hydroxyurea versus placebo. The working hypothesis is that children with SCA treated with hydroxyurea will have more medication-related hematologic toxicities, such as neutropenia, but no increase in SCA-related AEs (eg, pain crises, hospitalizations, requirement of blood transfusion) than children treated with placebo. We will test this hypothesis by comparing hematologic toxicities and AEs in children with SCA in the hydroxyurea versus placebo treatment groups. The frequency and incidence of each of the SCA-related hematologic toxicities listed in “Dose Toxicities” will be compared between the hydroxyurea and placebo groups using the chi-square test and negative binomial regression, respectively. Fractions of children having 1 or more AEs will be compared between hydroxyurea and placebo groups using chi-square tests, and incidence of all AEs per child will be compared between groups using negative binomial regression (because of overdispersion, as noted under Specific Aim 1). Secondary analysis will assess incidence and frequency of individual AEs.

*Specific Aim 3* is to define the relationship between hydroxyurea treatment and HbF, sICAM-1, and NO levels and between levels of these factors and risk of subsequent malaria. The working hypotheses of this aim are that (1) hydroxyurea will increase HbF and plasma NO levels and decrease plasma sICAM-1 levels and (2) HbF and plasma NO levels will inversely correlate, and plasma sICAM-1 levels will positively correlate, with subsequent malaria incidence. We will test this hypothesis by testing the association between (1) hydroxyurea and change in levels of HbF, sICAM-1, and NO at 2-, 4-, and 12-month follow-up and (2) change in levels of HbF, sICAM-1, and NO at 2 and 4 months and risk of subsequent malaria.

The hypothesized causal pathway is hydroxyurea → marker changes → increased malaria risk. To assess the first step in the causal pathway, the hydroxyurea and control groups will be compared according to change in and absolute level of HbF, sICAM-1, and NO levels. Markers will be analyzed separately; for each marker, the analysis will use a mixed linear model with child as the random effect and with fixed effects being group (hydroxyurea versus control) and visit (2, 4, and 12 months), possibly after transforming the marker, so, its distribution is more symmetric. Analysis for the second step in this pathway, association between change in or absolute level of HbF, sICAM-1, and NO levels and malaria risk, will use negative binomial regression as in Specific Aim 1. Each marker will be analyzed separately, and then, all 3 will be analyzed simultaneously. The predictors will be change in child's 2-month and 4-month marker measurements, and the outcome will be the child's counts of malaria episodes in the 10-month period between 2 and 12 months of follow-up, with adjustment for age, gender, local council area or village, and SES. For these analyses, hydroxyurea and placebo groups will be analyzed separately. Analysis of secondary predictors will be done analogously.

All analyses will be done as randomized analyses. Missing data will be recorded as missing and will not be used in analysis.

### Duration of Study Participation

The primary study end point will be evaluated after 12 months of study treatment (hydroxyurea or placebo). After these 12 months, children will enter a follow-up phase, during which they can receive an additional 12 months of open-label hydroxyurea treatment (through the full period of study follow-up) if they or their parents wish to do so, after consultation with local physicians at the MHSCC, and a clear explanation of the potential benefits and problems of treatment with hydroxyurea. We are working with the Ministry of Health to see if hydroxyurea can be subsidized, if it is safe and effective, so that children can continue to receive it after the trial is completed. Parents or guardians who are concerned that their child suffered harm from study participation are encouraged to talk to study personnel, who will review the issue with the ethics review committee and determine whether compensation is required.

### Monitoring

Hydroxyurea is the only disease-modifying therapy that is approved by the Food and Drug Administration (FDA) for SCA [35,36]. Approval was based on a landmark randomized trial, by Charache et al, which showed decreased episodes of acute chest syndrome, painful crises, blood transfusions, and hospitalizations among adults with SCA [37]. Hydroxyurea is not yet approved by the FDA for pediatric SCA patients. However, various studies of children with SCA have demonstrated its clinical and laboratory efficacy, in addition to its safety [8,38,39].

Hematologic toxicities and occasional dose adjustments are expected with hydroxyurea therapy. Laboratory toxicities from hydroxyurea will manifest primarily as reversible and transient myelosuppression, especially of granulocytes [40]. Careful monitoring of complete blood counts will be performed at 2 weeks and then at 1, 2, 3, 4, 6, 8, 10, and 12 months after initiation of study treatment. If a hematologic toxicity occurs (eg, the ANC falls <1.0 × 10^9^/L), study treatment will be held and weekly blood counts performed. If the hematologic toxicity resolves within 2 weeks, the daily dose will resume at 20 ± 2.5 mg/kg/day. If the toxicity persists for 2 weeks or occurs twice within a 3-month period, the study treatment dose will be reduced by 5 mg/kg/day to 15 ± 2.5 mg/kg/day.

A similar algorithm will be used for platelet toxicity, defined as <80 × 10^9^/L. For anemia, the thresholds will be any of the following: if the Hb concentration falls to <4.0 g/dL; or Hb <6.0 g/dL with the ARC <100 × 10^9^/L; or Hb <7.0 g/dL with the ARC <80 × 10^9^/L, study treatment should be withheld until weekly counts document recovery. If the toxicity resolves within 2 weeks, the daily dose will resume at 20 ± 2.5 mg/kg/day. If the toxicity persists for 2 weeks or occurs twice within a 3-month period, the study treatment dose will be reduced by 5 mg/kg/day to 15 ± 2.5 mg/kg/day. Study treatment will also be held during acute hepatic or renal toxicity (eg, ALT >2× the upper limit of normal for age, creatinine more than doubled from the baseline value, and >1.0 mg/dL).

An independent DSMB will supervise the trial. The DSMB will consist of a group of experienced investigators and a lay advocate. Among these individuals, there will be expertise in international pediatric hematology clinical research and specifically malaria and the use of hydroxyurea in SCA, as well as representation in biostatistics, ethics, clinical research, and patient advocacy.

In our trial, we expect AEs to occur, but we expect that children with SCA treated with hydroxyurea will have more medication-related hematologic toxicities, but no increase in SCA-related AEs. After obtaining written consent from study participants, all AEs and serious adverse events (SAE) will be collected and reported to the MCC and the DCC using the correct data collection forms. The Common Terminology Criteria for Adverse Events version 4.0, available since 2009, will be used for AE reporting. All AEs are categorized by organ system and graded by severity. SAE reporting will occur in a timely manner to the DSMB and to the ethical boards that approved this study. All SAEs will be followed until resolution or stabilization.

Periodic review of data will occur with the external DSMB, and the NOHARM study will be discontinued if at any time the DSMB or study team feels that it is in the best interests of the study subjects. Because NOHARM is a phase III trial in a potentially vulnerable patient population, it is important that an independent group have access to the feasibility, safety, and efficacy data. Review by the DSMB will be critical to ensure that the subjects are protected from harm, while also ensuring that the study integrity is not compromised.

### Interim Analysis

Interim analyses will occur when 50, 100, and 150 participants have completed 1 year of follow-up (ie, the first analysis will occur when there are 25 children in each treatment arm, the next when there are 50 children in each treatment arm, and so forth). Clear stopping rules, presentation of data to the DSMB, and regular meetings of the DSMB will ensure that the NOHARM trial does indeed protect its study participants from harm, by determining whether differences in severe malaria or death rates between the 2 treatment arms are large enough to demonstrate superiority of one treatment arm over the other. Stopping rules will be created to evaluate: (1) excess risk of severe malaria (malaria requiring admission) and (2) the excess risk of death (the primary unexpected AE) in one treatment arm versus the other. The DSMB will review the protocol, including stopping rules, and decide on a schedule of meetings.

Each of the 2 harm outcomes listed previously will be monitored using a stopping rule based on the Lan–DeMets alpha-spending function approach. For each outcome, the total alpha (type I error risk) will be 0.025, so that the total chance of a false-positive finding (combining the 2 outcomes) is the usual 0.05. The stopping rule will be 2 sided. Different choices of the alpha-spending function make different allocations of the type I error risk between earlier and later interim analyses. The best-known spending function, the O'Brien–Fleming boundaries, is conservative in the sense of requiring a very small *P* value at early interim analyses (ie, require a very large difference between groups). Other spending functions (eg, power family or Hwang-Shih-DeCani, H-S-D, family) require somewhat smaller differences between groups at early interim analyses compared with O'Brien–Fleming, at the price of requiring somewhat larger differences in later interim analyses. These other spending functions can also provide slightly greater statistical power. Table 2 summarizes the *P* values required to stop at the 3 planned interim analyses (labeled as Analysis 1, 2, and 3) and the final analysis (labeled as Analysis 4), for some candidate spending functions. These are also the risk of a type I (false positive) error at each analysis, if in fact, the groups do not differ in the chance of an AE (For any spending function, the 4 *P* values add to .025, the overall type I error risk.) See Table 2 for *P* values required to stop at each interim analysis.

**Table 2:** *P* values required to stop at each interim analysis^a^.

	O'Brien	Pfam	H-S-D
Analysis	Fleming	Φ=3	γ=-3
1	<.001	<.001	.001
2	<.001	.003	.003
3	.007	.007	.007
4	.02	.01	.01

^a^The power family of spending functions has an adjustable constant Φ > 0; Φ=0 results in equal *P* values at all interim analyses, increasing Φ gives higher *P* values for later analyses. The H-S-D family has an adjustable constant γ≠ 0; γ=−4 gives approximately the O'Brien–Fleming spending function, whereas γ=1 results in approximately equal *P* values at all interim analyses.

To maximize the power for early detection of excess risk without unduly sacrificing power at the final analysis (Analysis 4), we prefer the H-S-D spending function with γ=−3.

### Progress to Date

**Figure 1:**
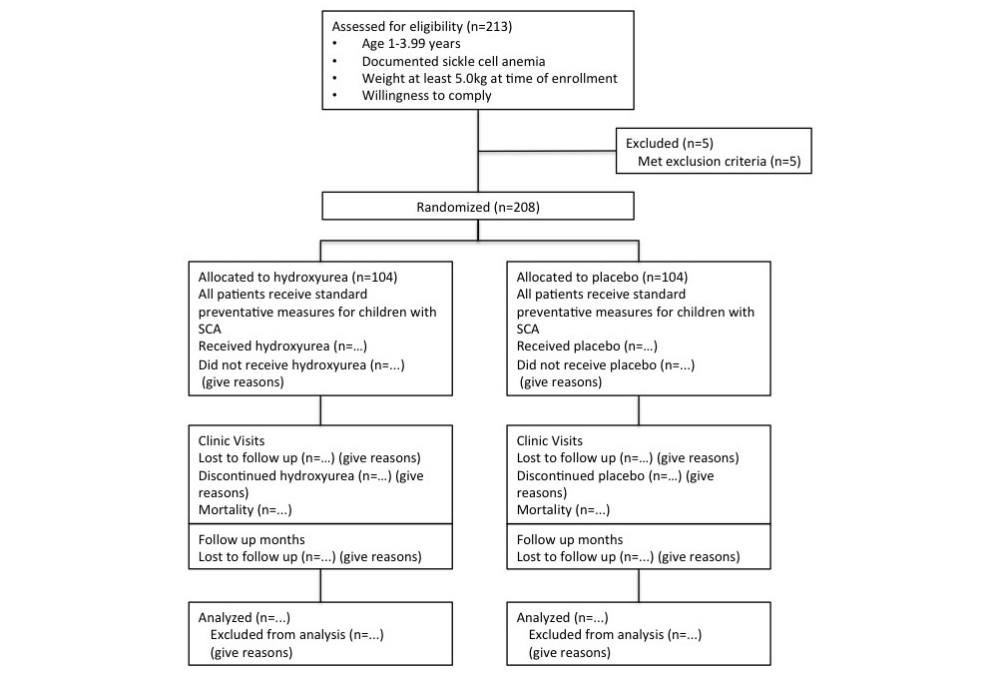
Participant flow diagram for the NOHARM study.

The NOHARM trial enrolled its first participants in September 2014, and enrollment was completed in November 2015. A total of 213 consented participants were enrolled, of which 208 were randomized into the blinded study treatment phase (months 0-12, Figure 1). As of March 28, 2016, 90 participants have completed the blinded treatment phase of the study, and monthly visits are currently ongoing. Data validation efforts are focused on the first cohort of participants to complete the blinded treatment, to ensure timely data analysis. The blinded study phase is scheduled to be completed in November 2016.

### Ethical Considerations

The NOHARM study received ethical approval from Makerere University School of Medicine Research and Ethics Committee (SOMREC; Kampala, Uganda; approval #2014-006), the Mulago Hospital Research and Ethics Committee (approval #528), the University of Minnesota Institutional Review Board (approval # 1310M44703), Indiana University Institutional Review Board (Bloomington, IN, USA; approval #1412985992), Cincinnati Children’s Hospital Medical Center (approval #2014-1409), the Uganda National Council on Science and Technology (approval #HS 1553), and the Uganda National Drug Authority. In the event of the need for protocol amendments, such changes will be discussed with all investigators on the trial, and once agreed on, a protocol amendment form or a change in protocol request will be made to the appropriate IRB or ethics committees.

Informed consent is obtained after discussion between at least 1 parent or guardian of a prospective study participant and trained study team personnel. Every consent discussion will include: purpose of the study, study procedures (including enrollment, screening, randomization, treatment, monitoring, and follow-up), and potential benefits or risks of hydroxyurea. An additional consent for sharing and storing blood samples is also obtained. All discussions and informed consent processes will be completed in either English or Luganda, at an education-appropriate level.

### Dissemination

When the open-label phase is completed and the study analysis done, we will provide a forum for all interested study participants to review the study findings. We will review them in clear, nontechnical terms in the local language and leave time for questions and ongoing discussion after the session. The final study findings will be published in a peer-reviewed publication and presented at 1 or more national or international meetings, including meetings in Uganda. We will use standard International Committee of Medical Journal Editors criteria for authorship. Through this publication, the public will have access to the study protocol. Participant level data will be maintained in our database until fully analyzed. If permitted by the study IRBs, we will eventually make participant-level data available to researchers on request in a deidentified dataset.

## Results

As noted in the *Progress to Date* subsection of the *Methods* section, as of March 28, 2016, 90 participants have completed the blinded treatment phase of the study, and all study participants are expected to have completed the blinded treatment phase of the study by November 2016. We anticipate analysis of results for the blinded phase to be completed by early to mid-2017. The open-label phase of the study will be completed in November 2017, and we are working with the Ministry of Health and other partners to see how children in the study can continue hydroxyurea if it proves to be safe and effective (Multimedia Appendix 5).

## Discussion

Hydroxyurea as a once-daily oral medication has been shown to be safe, well tolerated, and easy to administer. Pivotal trials, such as the Phase III BABY HUG trial, have shown the clinical benefits of hydroxyurea in very young children [8]. In addition, hydroxyurea is associated with decreased medical care costs. A recent study in pediatrics by Wang et al looked at the children from the BABY HUG trial and estimated the cost of medical care for those taking hydroxyurea versus those taking placebo and found that the total yearly cost for children on hydroxyurea was estimated at $11 072, which was 21% less than the placebo group, whose annual costs averaged $13 962 [41]. If hydroxyurea is safe and effective for children with SCA in Uganda, advocacy for subsidization of the cost will be explored. Hydroxyurea is available in Uganda and is used in some private clinics for children with SCA, but the drug is not yet registered by the National Drug Authority, and there is no formal Ministry of Health approval for this indication, so, it is not routinely provided to children in the MHSCC. In addition, if this study shows that hydroxyurea is beneficial, clinicians at the MHSCC will have received training and gained valuable experience that will enable them to assume the provision of care and monitoring of children with SCA who are receiving hydroxyurea.

Conducting a trial in a resource-limited setting poses some challenges in regard to ethical considerations and informed consent processes. However, the challenges faced with regard to participant or guardian understanding of clinical trials are not unique to Sub-Saharan Africa [42,43]. Multiple papers discuss ethical considerations of doing trials in Sub-Saharan Africa, highlighting issues such as differences in health care systems, limited access to care, quality of informed consent, educational disparities, and knowledge of research, as factors that could make participants vulnerable to exploitation [44-47].

Concerns about the misunderstandings parents may have with collecting blood samples and the use of samples for future research are also an important consideration for our trial. Studies in resource-limited countries highlight some of the misgivings participants have in relation to blood [48-50], ranging from the volume of blood being taken from an already sick child to concerns that the blood will be sold and used in witchcraft [51,52].

The vulnerability of our study population, which is well documented in the literature, adds another dimension to the trial [53-55]. In addition, the phenomenon of therapeutic misconception, where research participants fail to differentiate the consequences of research participation from ordinary treatment, is also well described in the literature, and is another challenge, especially in our cross-cultural setting [56-58].

For this trial, we received local ethics approval from the SOMREC, at Makerere University College of Health Sciences before trial commencement (in addition to approval from multiple other institutions listed in the *Ethical Considerations* section). A culturally sensitive, language-appropriate informed consent has been developed and aligns with the format recommended by the SOMREC. Aside from the ethical challenges, ensuring adequate laboratory infrastructure has been essential, especially with the frequency of blood draws in our study. Qualified laboratory staff performed blood draws, and topical anesthetics were used to minimize discomfort related to these procedures.

One limitation of this trial pertains to our ability to assess the primary end point, namely, the effects of 12 months of hydroxyurea versus placebo treatment on malaria incidence. We will provide ITNs and standard malaria prophylaxis to all NOHARM study children, which should lower the incidence of malarial infections, and thus could potentially impact our primary end point. Prevalence data obtained for sample size calculations were collected from populations in which children did receive ITNs; however, these populations may live in areas with higher malaria incidence because some children in those neighborhoods developed severe malaria. If malaria incidence in the study population is low, we may have power to detect only very large differences in malaria incidence between the treatment and placebo groups. In this circumstance, valuable data will still be gained about safety, incidence of neutropenia and infections other than malaria, and effectiveness against vascular crises and other complications of SCA. We will be able to provide estimates of the differences in malaria incidence, but these estimates will have wide confidence intervals, so, further study in areas of higher malaria transmission may be required to determine more precisely the risk of malaria and severe malaria with hydroxyurea treatment in SCA.

Other potential limitations pertain to randomization and blinding. Ideally, persons treating study participants would be separate from the trial, so that changes in laboratory values will not lead study personnel to guess the study arm in which the child is enrolled. In this study, to minimize the potential confusion of having multiple physicians and nurses involved in evaluation and care of the child, we instead instituted a system where laboratory results for all scheduled visits came to the data manager, and the clinicians taking care of the child saw laboratory values only if they were critical values. Thus, most laboratory values were not seen by clinical study personnel and made it unlikely that they would or could accurately guess the study arm for most study children.

In summary, the NOHARM trial is a randomized controlled clinical trial of hydroxyurea for children with SCA in a malaria-endemic region. If the study demonstrates a clear benefit of hydroxyurea with no increased malaria risk or AEs in children with SCA, these critical data could lead to transformation of treatment practices for SCA across Africa.

### Trial Status

The trial is currently ongoing. Children are currently in either the randomized treatment phase or open-label treatment phase of follow-up.

## Appendix

Multimedia Appendix 1 Participant timeline.

Multimedia Appendix 2 Informed consent materials.

Multimedia Appendix 3 Makerere School of Medicine Research Ethics Committee Comments and investigator replies.

Multimedia Appendix 4 University of Minnesota IRB peer review comments and investigator responses.

Multimedia Appendix 5 Spirit Checklist.

## Abbreviations

AE: adverse event
ALT: alanine aminotransferase
ANC: absolute neutrophil count
ARC: absolute reticulocyte count
CTCAE: Common Terminology Criteria for Adverse Events
DCC: data coordinating center
DSMB: Data Safety and Monitoring Board
FDA: Food and Drug Administration
Hb: hemoglobin
HbAS: hemoglobin AS
HbF: fetal hemoglobin
ICAM-1: intracellular adhesion molecule-1
IRB: institutional review board
ITN: insecticide-treated bednet
MCC: Medical Coordinating Center
MHSCC: Mulago Hospital Sickle Cell Clinic
NO: nitric oxide
NOHARM: Novel use Of Hydroxyurea in an African Region with Malaria
SAE: severe adverse event
SCA: sickle cell anemia
SES: socioeconomic status
sICAM-1: soluble intracellular adhesion molecule-1
SOMREC: School of Medicine Research and Ethics Committee
TNF: tumor necrosis factor
VCAM: vascular cell adhesion molecule
VWF: von Willebrand factor
WHO: World Health Organization
