# Supplementary material for: Novel Use of Hydroxyurea in an African Region With Malaria: Protocol for a Randomized Controlled Clinical Trial
Source: JMIR Res Protoc. 2016 Jun 23;5(2):e110. doi: 10.2196/resprot.5599 (PMC4937184; doi:10.2196/resprot.5599)
Supplement: Multimedia Appendix 2 [file resprot_v5i2e110_app2.pdf]

CONSENT FORM

A. Introduction

You are being invited to participate in this research study because your child has Sickle Cell Anemia (SCA). Please read or listen to this information and ask any questions before you agree to take part in this study. If you understand and agree to take part in this study, we will ask you to sign this consent form.

This study is being conducted by Prof. Christopher Ndugwa, Dr. Phillip Kasirye and colleagues at the Department of Paediatrics, Mulago Hospital in collaboration with Prof. Chandy John and colleagues from the Indiana University, USA.

B. Purpose

This study is to test whether children taking hydroxyurea for SCA have more risk of malaria than those not taking hydroxyurea. Hydroxyurea is a medication that is used to treat SCA in other areas of the world, but it has not yet been studied in children with SCA who live in areas where there is malaria. This study will help us understand whether hydroxyurea is safe for these children. In this study children will be divided by chance to get either hydroxyurea or a placebo. (A placebo is a tablet that looks like hydroxyurea but contains no medication).

C. Study Procedures

The specific study procedures are:

1. **Enrollment**
   There will be one year of study treatment and then a year of follow-up. During the year of follow-up, participants may decide to take hydroxyurea after discussing the potential benefits and risks with a doctor.

2. **Screening**

   A child needs to be healthy enough to participate in this study, so one of the first things we shall do is examine (screen) your child to make sure s/he is fit for participation. Screening will involve doing a physical examination of your child and some blood tests. About 2 teaspoons (10mls) of blood will be drawn to check for malaria, HIV, blood cell counts and levels of blood substances that tell us how your child's liver and kidneys are working.

   If there is a delay in getting the results of these tests, we may ask you to return to the clinic 1-3 days later to get them.

   200 children who are found to be healthy enough to participate in the study will go on to randomization. If your child in not healthy enough to participate in the study, your child will first need to receive treatment at the MHSCC or Mulago Hospital. Once your child has recovered, we will evaluate them again (using the same procedures described above) to see if they can be part of the study.

3. **Randomization**

   Randomization means we shall decide by chance whether your child will receive hydroxyurea or the placebo. A computer program run by someone who is not directly involved in the study will determine the type of medicine your child will take. Your child will have an equal chance of receiving either medicine. Since the two types of medicine look exactly the same, neither you nor the study team will know what group your child is in until your child completes the study or withdraws from the study.

4. **Treatment**

   Your child will be required to take the study treatment assigned to them (either hydroxyurea or placebo) for 1 year. The medicine is given in tablet form and will be taken once every day.

   After your child has completed 1 year of study treatment, there will be at least 1 year of follow-up. During this follow-up period, you may decide to let your child take hydroxyurea after a discussion with the doctor about the possible benefits and risks of taking the medicine.

   During this study, your child will continue to receive all the regular care and medications that are provided by the sickle cell clinic.

5. **Monitoring and follow-up**

If you agree to let your child participate in this study, you must bring the child to the clinic for every scheduled study visit. These visits are important because they will help us monitor how your child is responding to the medicine and will allow us to check if your child has malaria. There will be 10 visits during the first year (every two weeks for the first month, then monthly until month 4, then every two months until the end of the first year).
In the second year, there shall be 2 visits for everyone (one at 18 months and one at the end of the second year). However if you opt to have your child take the open label hydroxyurea, we shall need to see your child every 2 months to monitor him or her. This means you must bring your child for a clinic visit at 14, 16, 18 ,20, 22 and 24 months, for a total of 6 visits during the year. During each of these visits we will dispense hydroxyurea and take a small amount of blood to monitor blood counts to ensure your child is safe taking the medicine.
If you do not wish to have your child take the open label hydroxyurea, we shall have only 2 visits in the second year.

The study team will regularly remind you and help you keep track of visit dates.

During each visit, we shall:

a. *Ask some questions* to see how your child's health has been.
b. *Do a physical examination* to assess your child's health.
c. *Draw blood*: For most visits, we will need just ¼ teaspoon (1 ml) of blood. For Visits 1, 4, 6, 10, 12, 18, and 24 we will need more blood, but we will never take more than 2 teaspoons (10 ml) of blood from you at any visit. A trained person will draw the blood from your child's vein. Our tests will look for malaria and we will also look at blood cell counts, types of hemoglobin (the substance in blood that carries oxygen) and different factors in blood. These tests may help us understand how children with SCA do when taking hydroxyurea.
d. *Make sure your child is taking the study medication properly.*

6. The Open label phase in year 2

In the second year all study children will be given the option to be on hydroxyurea. You may therefore choose not to have your child take hydroxyurea.

If you choose to let your child take the hydroxyurea, you may stop it at any time if you choose to do so. However, if you do not choose to start on open-label hydroxyurea at this time, we will not be able to place your child on hydroxyurea later in the study period.

7. We also ask that you bring your child to the Sickle Cell Clinic any time the child has an illness. We will record information about your child's illness, because we need to know if it might be related to the drug treatment or to sickle cell disease. All clinical care required for your child will be provided free of charge for the duration of the study.

D. Potential Risks

Hydroxyurea Therapy: The most common potential side effect of hydroxyurea is that it may lower the number of white blood cells, red blood cells, and platelets. We shall regularly check the blood counts to make sure they do not reach dangerous levels that can cause problems. The medicine will be stopped for a week or two if the blood counts ever get too low.

Other, less common, side effects of taking hydroxyurea may include: darkening of the skin or nails; stomach upset or headache; fever, rash, hair loss, or dizziness.

Your doctors will tell you if they learn new things from this or other studies that may affect your child's health, safety or your willingness for your child to stay in this study.

Blood Draws

When we take a blood sample, the needle hurts a little and may cause some bruising on the arm. There is a very small chance that one may get an infection where the needle went in. We have not had this problem in drawing blood from thousands of children in previous studies, but if infection does develop, we will treat it.

E. Potential Benefits

Your child will receive no direct benefit from participation in this study. His/her participation may help the investigators find out whether

hydroxyurea works for children with SCA in areas where malaria is common. If we find that this is true, then this study will give us better ways to treat SCA in areas where there is malaria and will potentially improve the lives of many children.

F. Re-imbursements and Compensations:

All clinical care required for your child will be provided free of charge for the duration of the study. You will be reimbursed for the cost of transportation to and from all study visits. In addition, we will provide Uganda shillings 10,000/= to compensate you for your time and effort while on this study. If your child becomes ill and needs to travel back to Mulago Hospital for tests or for treatment, your travel expenses will be refunded.

G. Alternatives to Study Participation

Hydroxyurea is not yet recommended for use in Uganda in the routine treatment of sickle cell anemia. We are not aware of any appropriate alternative treatments available in Uganda to treat and manage children with SCA. Your child will continue to receive all the routine care provided by the MHSCC and Mulago Hospital regardless of whether he/she is in this study.

H. Research Related Injury

Under some circumstances the sponsor of the study will pay for care for injuries resulting directly from being in the study. If you want information about those circumstances or if you think you have suffered a research related injury let the study doctors know right away.

I. Voluntary Nature of the Study

You may choose not to let your child participate in this study. Your decision whether or not to have your child participate in this study will not affect your current or future relations with Mulago Hospital or the Mulago Hospital Sickle Cell Clinic. If you agree to have your child participate in the study but later change your mind, you are free to withdraw your child at any time and will not lose any services, rights or benefits to which you or your child are otherwise entitled. If you want to leave the study, call **Dr. Philip Kasirye** on 0784560799 to let us know.

J. Confidentiality

The records of this study will be kept private. In any publications or presentations, we will not include any information that will make it possible to identify you or your child as a subject. Your child's record for the study may, however, be reviewed by the ethical and regulatory bodies who have authorized this study. Your child's confidentiality will be protected to the maximum extent allowable by law.

K. Contacts and Questions: For questions related to this study are you are encouraged to speak to **Dr. Philip Kasirye** on 0784560799 or **Dr. Robert O. Opoka** on 0772996164.

L. Participant rights

If you have any questions or concerns regarding the study and would like to talk to someone other than the researcher(s), you are encouraged to contact **Prof. James Tumwine**, Chairman of the Makerere University School of Medicine Research and Ethics Committee (SOMREC), on 0414530020.

M. Statement of Consent

I have read or listened to the above information. I have asked questions and received answers.

1. I consent to participate in the study.

☐ Yes ☐ No

2. I would like my child to take hydroxyurea during the second year of the study and agree to the additional visit and blood draw requirements.

☐ Yes ☐ No

Printed Name of Research Participant

Signature or thumbprint of Parent or Guardian Date

Printed Name of an impartial witness (Applicable where Parent or Guardian is illiterate)

Signature of a witness (if Parent or Guardian is illiterate) Date

Name and Signature of Person Obtaining Consent Date
