# Supplementary material for: Novel Use of Hydroxyurea in an African Region With Malaria: Protocol for a Randomized Controlled Clinical Trial
Source: JMIR Res Protoc. 2016 Jun 23;5(2):e110. doi: 10.2196/resprot.5599 (PMC4937184; doi:10.2196/resprot.5599)
Supplement: Multimedia Appendix 3 [file resprot_v5i2e110_app3.pdf]

January 8, 2014

To: Prof. James Tumwine
Chairperson, SOMREC

RE: Response to SOMREC comments and suggestions on Dr. Christopher Ndugwa's study entitled "Novel use of hydroxyurea in an African region with Malaria (NOHARM)"

We would like to thank the committee for reviewing our study and for all the useful comments and suggestions. We would like respond to your feedback as follows:

- Ethical considerations-the patients are denied of receiving the valuable treatment, which is internationally considered the standard of care since it is believed to be beneficial to them. This poses an ethical problem.

  The primary focus of this study is not to study whether hydroxyurea (HU) is beneficial in preventing vaso-occlusive crises in children with sickle cell anemia (SCA), but to study whether it increases or decreases the risk of malaria in these children. As stated in the protocol, there have been no randomized clinical trials of hydroxyurea in children with SCA in malaria endemic areas of Africa. Children with SCA are more likely to die if they get malaria than children without SCA; so, malaria in SCA is a serious concern. We believe hydroxyurea is likely to be beneficial in preventing vaso-occlusive crises, as it has been elsewhere, but there are reasons to believe it might increase the risk of malaria, through an increase in ICAM-1 expression, which can increase malaria parasite binding. However, it is also possible that the increase in hemoglobin F that occurs with HU treatment could decrease the risk of malaria. Without a clinical trial, we will not know whether HU increases or decreases the risk of malaria. If HU increases the risk of malaria and severe malaria, that risk could outweigh the potential benefit of reduced vaso-occlusive crises.

  In addition, where HU is used, it has had to be closely monitored. The toxicities of HU in malaria endemic areas of Africa have also not been studied. For example, it is known that HU can lead to neutropenia. The increased risk of infection with neutropenia could be greater for children with SCA in our setting than in the areas of North America and Europe where HU has been studied in clinical trials.

  These potential risks of HU treatment make it clear that a randomized clinical trial is necessary, as without such a trial, children receiving HU may be exposed to significant risk. This study is being conducted because we do not yet know if HU will be beneficial in children with SCA in sub-Saharan Africa living in a malaria endemic area. If the risk of malaria and severe neutropenia is increased, hydroxyurea may not be beneficial. That is why we propose to conduct this study.

  Sections 1.1 to 1.6 of the protocol provide a more comprehensive overview of the points we have stated above. Section 1.6 in particular emphasizes our reasons for doing a placebo-controlled trial. (see pages 6-10)

- It is not clear how you will ensure that the benefits are continued even after the study. You need to state that clearly.

  1) Addmedica has agreed to donate enough HU to supply children for an additional two years after the trial has been completed, if it is found to be beneficial.

  2) Hydroxyurea is already on the essential drug list in Uganda, but is listed as a drug for cancer patients. We have had discussions with the MOH and the MOH has indicated that if found to be beneficial, they are ready to make HU available for use for children with sickle cell anemia in Uganda.

  3) It must be noted that patients receiving HU need to be closely monitored for possible side effects of HU. Making HU available for children with SCA in Uganda will need to be accompanied by training of clinicians and the development of protocols that will enable clinicians to closely follow these patients. In this respect, our group will work closely with the sickle cell clinic in Mulago so that in the long run they can take over the provision of care and monitoring of children with SCA who are receiving HU.

  Sections 1.6 and 1.7 of the protocol outline the long-term benefits of the study. We have amended Section 1.7 to state these benefits more clearly. (see pages 10-11)

- Capacity building –To what extent will the study benefit the science world and the institution at large?

  1) If HU is shown to be safe in malaria endemic areas in terms of not increasing malaria risk, and if it is as effective in reducing vaso-occlusive crises as in non-malaria endemic areas, then it can be safely introduced in malaria endemic areas. Data on the other risks of HU in these areas (e.g., neutropenia, anemia) will also be important in informing other malaria endemic regions in low to middle income countries about what they need to monitor, and how often. The study could transform care of children with sickle cell disease in sub-Saharan Africa.

  2) Our group will work closely with the sickle cell clinic in Mulago to train nurses, physicians and laboratory personnel so that in the long run they can take over the provision of care and monitoring of children with SCA who are receiving HU.

  These details have been included in Section 1.7 of the revised protocol. (see pages 10-11)

- You should include a detailed schedule of the DSMB and the stopping rules

  We thank you for this comment and have revised Section 9.3 of the protocol to clarify the schedule of the DSMB and the stopping rules as suggested. (see pagess29-30)

  In brief …
  The DSMB will review the protocol, including stopping rules, and decide on a schedule of meetings, but for the present purpose we propose meetings of the DSMB and interim analyses when 50, 100 and 150 participants have completed 1 year of follow-up up (i.e. the

first analysis will occur when there are 25 children in each treatment arm, the next when there are 50 children in each treatment arm etc.).

Stopping rules will evaluate (1) excess risk of severe malaria (malaria requiring admission), (2) the excess risk of death (the primary unexpected adverse event) in one treatment arm versus the other.

Each of the two harm outcomes listed above will be monitored using a stopping rule based on the Lan-DeMets alpha-spending function approach. For each outcome, the total alpha (type I error risk) will be 0.025, so that the total chance of a false-positive finding (combining the two outcomes) is the usual 0.05. The stopping rule will be two-sided. Different choices of the alpha-spending function make different allocations of the type I error risk between earlier and later interim analyses. The best-known spending function, the O'Brien-Fleming boundaries, is conservative in the sense of requiring a very small P-value at early interim analyses (i.e., require a very large difference between groups). Other spending functions (e.g., power family [Pfam] or Hwang-Shih-DeCani [H-S-D] family) require somewhat smaller differences between groups at early interim analyses compared to O'Brien-Fleming, at the price of requiring somewhat larger differences in later interim analyses. These other spending functions can also provide slightly greater statistical power. Table X below shows the P-values required to stop at the three planned interim analyses (labelled as Analysis 1, 2, and 3) and the final analysis (labelled as Analysis 4), for some candidate spending functions. These are also the risk of a type I (false positive) error at each analysis, if in fact the groups do not differ in the chance of an adverse event. (For any spending function, the four P-values add to 0.025, the overall type I error risk.)

Table X. P-values required to stop at each interim analysis*

	O'Brien	Pfam	H-S-D
Analysis	Fleming	$\varphi = 3$	= -3
1	0.00000	0.00039	0.00146
2	0.00082	0.00273	0.00310
3	0.00703	0.00742	0.00656
4	0.01715	0.01445	0.01388

* The power family of spending functions has an adjustable constant $\varphi > 0$; $\varphi = 0$ results in equal P-values at all interim analyses, increasing φ gives higher P-values for later analyses. The Hwang-Shih-DeCani family has an adjustable constant ≠ 0; = -4 gives approximately the O'Brien-Fleming spending function, while = 1 results in approximately equal P-values at all interim analyses.

To maximize the power for early detection of excess risk without unduly sacrificing power at the final analysis (Analysis 4), we prefer the Hwang-Shih-DeCani (H-S-D) spending function with = -3.

Clear stopping rules, presentation of data to the DSMB, and regular meetings of the DSMB will ensure that the NOHARM trial does indeed protect its study participants from harm, by

determining if differences in severe malaria or death rates between the two treatment arms are large enough to demonstrate superiority of one treatment arm over the other.

- National drug authority does not consider Hydroxyurea as a study drug. How do you intend to work with national drug authority to get the study approved?

  We have submitted an application to the NDA and are currently working with them to get approval for the drug. If HU is found to be beneficial we shall provide the NDA with information that will enable them to work on mechanisms to approve HU for use in children with SCA in Uganda.

- What placebo will you use to compare against Hydroxyurea? Is there equipoise in sicklers as far as this drug is concerned? Make it clear in the proposal with appropriate references.

  We have included details of the placebo in Section 1.5 of the protocol as suggested. (see page 10)

  Briefly…
  The placebo will be made by the same manufacturer (AddMedica) as the hydroxyurea, will be identical in size and appearance, and will contain an inert cellulose necessary for tablet structure (Prosolv V SMCC 90 [silicified microcrystalline cellulose]), which is also an inactive component of the hydroxyurea tablets.

  The placebo is an inert, chemically inactive substance. The manufacturer has provided a Certificate of Analysis as well as a Good Manufacturing Practice (GMP) Certificate of Manufacture for this placebo which we have already submitted to the National Drug Authority.

  We have already explained earlier that we think there is equipoise as far as the use of HU is concerned and provided references for this in the proposal.

- What standard will be used to address adverse events? How are you going to report them?

  Adverse events (AE) reporting will utilize the Common Terminology Criteria for Adverse Events (CTCAE) Version 4.0 available since 2009, where all AE are categorized by organ system and graded by severity. Please refer to Appendix III of the study protocol for a list of expected and potentially serious AE, as well as exceptions to the CTCAE.

  Serious adverse events (SAE) reporting will use commonly accepted definitions (any life-threatening condition or death) with the exception of hospitalization, which is common in children with SCA. At Mulago Hospital, children who are admitted with sickle cell-related conditions (such as anemia requiring transfusion, acute chest syndrome, and stroke) have an average length of stay of approximately 7 days. Accordingly, hospital stays must be more than 7 days to be scored as SAE in NOHARM. SAE for this study will therefore be defined as 1) hospitalization for more than 7 days; 2) any other life-threatening condition; or 3) death. Drug-related and unexpected SAE are of particular concern, however all SAE including death require prompt reporting by the local site, using email communication or telephone. If an ongoing SAE changes in its intensity or relationship to the investigational product, a follow-

up SAE report should be sent to the MCC and DCC. All SAE will be followed until resolution or stabilization. Unexpected SAE (deaths) will be reported to the DSMB and IRBs within 7 days of the team being aware of the death. Other SAE will be reported to the DSMB and IRBs in the 6- monthly (DSMB) or annual (IRB) study reports to these organizations.

Section 4.8 of the protocol highlights the details of how adverse events reporting will occur. (see pages 16-17)

- Please clarify on the Block randomization to be used.

  We shall be doing randomization in blocks of 8 or more. In this way, it will be impossible to predict the allocation of patients as they are enrolled into the study. Children will be randomized into treatment groups by order of entry in the study, based on a pre-determined blinded randomization list. The child's study identification number will be recorded and treatment group may only be determined by comparing the child's study id to the blinded list, which only the study pharmacist and the DCC staff will have access to until the study is completed or stopping rules are reached and unblinding is required.

  The above detail has been added to Section 7.2 of the protocol to make it clear. (see page 25)

- The fixed sample size at 100 needs to be revised for a clinically significant difference of at least 25%. The differences of objective used for objective 1 and 2 are quite high.

  We thank you for this comment and it is in our interest not to expose more patients than is necessary to the effects of HU.

  With 100 children per group, the study has 90% power to detect 34% difference between groups respectively in malaria incidence, and 80% power to detect a 25% difference between the study groups. Thus the study with this sample size does have power to detect a 25% difference with the standard and accepted study power of 80%. The stopping rules are designed such that if there are significant differences in harm outcomes (i.e. risk of severe malaria or risk of death) at 25, 50 or 75 children enrolled per group (HU vs. placebo), or 50, 100 or 150 children enrolled total, the study will be stopped. We have done this to ensure that statistically notable differences between groups in significant adverse outcomes, even at very early stages of the study, will end the study, so that children are not put at risk for these adverse events.

  So, although we have a sample size of 100, the stopping rules are in place to ensure that the study will be stopped as soon as there is evidence of effect, which can be as early as 50 patients enrolled. We did not want to do such an important study and end up not conclusively proving effect because of low power.

  The stopping rules are detailed in Section 9.3 of the protocol. (see pages 29-30)

- You need to exclude patients who can afford Hydroxyurea.

This point is well taken. We will exclude patients who are on already on hydroxyurea and are taking it regularly.

We have added this detail to Section 3.2 of the protocol. (see page 12)

- Block randomization with what size of blocks?
  We shall be doing randomization in blocks of 8 or more as clarified above.

Again, we thank you for your constructive feedback. We hope that the responses, changes and explanations made are satisfactory. We believe that this is going to be one of the landmark studies on hydroxyurea and that it will provide conclusive evidence on the suitability of using hydroxyurea in malaria endemic regions of sub-Saharan Africa.

We will be happy to respond to any other comments and suggestions that you might have about this study.

Thank you.

Sincerely,

Prof. Christopher Ndugwa

Principal Investigator
