# Supplementary material for: Novel Use of Hydroxyurea in an African Region With Malaria: Protocol for a Randomized Controlled Clinical Trial
Source: JMIR Res Protoc. 2016 Jun 23;5(2):e110. doi: 10.2196/resprot.5599 (PMC4937184; doi:10.2196/resprot.5599)
Supplement: Multimedia Appendix 4 [file resprot_v5i2e110_app4.pdf]

Response to IRB Action for study number 1310M44703, “Novel use of Hydroxyurea in an African Region with Malaria (NOHARM): A Randomized Double-Blinded Placebo Controlled Trial for Children with Sickle Cell Anemia (NOHARM).”

Additional Requirements Pending Approval:

1. The committee determined that insufficient information was submitted to confirm if the study drug (Siklos/Hydroxycarbamide) is Investigational Drug Number (IND) exempt, and will not require oversight by the Food and Drug Administration (FDA). Please provide conformation from the FDA that an IND is not required.

   Response: Find FDA exemption letter attached.

2. Please confirm the Principal Investigator, Chandy John, and all of the Co-Investigators/Staff that are located within the United States have completed HIPAA training.

   Response: We confirm that Dr. Chandy John and all US-based study personnel have completed HIPAA training.

3. Please provide documentation of approval from cooperating site Makerere University School of Medicine Research and Ethics Committee (SOMREC), as indicated in item 3.5 (Is this research being reviewed by any other institution or peer review committee) of the application.

   Response: Find SOMREC approval documentation attached. Please note that SOMREC required minor changes to our protocol, and the revised protocol (version 1.1) is also attached, with changes highlighted in yellow in the Word document.

4. Item 7.5 (Will subjects receive inducements before or rewards after the research study to include reimbursement for travel or parking) of the application was selected to indicate "Yes," and was stated in the paragraph below that "All travel expenses will be reimbursed. At every study visit, participants will receive remuneration of 10,000 Uganda Shillings (approximately $4) for the time and effort to defray the cost of meals." Please add "(approximately $4)" to define the equivalency of 10,000 Uganda shillings and time and effort to "defray the cost of meals" to the "Re-imbursements and Compensation" section of the consent forms.

   Response: The consent forms will be used only by Ugandan personnel and study subjects, and we have been advised by Ugandan collaborators that adding the approximate US dollar equivalent may cause unnecessary confusion among study participants and staff. Ugandan shillings will be the currency used to compensate all study participants for their travel and efforts

   The approximation was noted in the application to the IRB for clarity for the IRB committee who are likely more familiar with USD as currency.

5. Please use the standard IRB consent form template design. Remove the consent form header from all the pages, starting on page two. The following items are now required to be included in the footer of each page of the consent form: study code number, correct pagination (page x of y), and consent form *version date*. Please add the study code number and update the version date to each page of the consent form, they sh ould be aligned to left margin in a column.

   Response: Find updated standardized IRB consent forms attached. Makere University prefers to have a header on each page, so the header has been retained on all pages.

In addition, the committee would like to make the following suggestions. If you wish to incorporate these suggestions, please include them on your revised submission:

1. The committee noted that the consent form for storing and sharing blood samples contained information regarding the primary study, and could be shortened if the content was removed.

   Response; The information regarding the primary study was placed there to satisfy Makerere University requirements, so we have maintained it in the form.
